# Effect of Coronary Sinus Reducer Implantation on Aerobic Exercise Capacity in Refractory Angina Patients—A CROSSROAD Study

**DOI:** 10.3390/jcdd10060235

**Published:** 2023-05-26

**Authors:** Miha Mrak, Nejc Pavšič, David Žižek, Luka Ležaić, Matjaž Bunc

**Affiliations:** 1 Department of Cardiology, University Medical Centre Ljubljana, 1000 Ljubljana, Slovenia; miha.mrak@kclj.si (M.M.); nejc.pavsic@kclj.si (N.P.);; 2 Faculty of Medicine, University of Ljubljana, 1000 Ljubljana, Slovenia; 3 Department of Nuclear Medicine, University Medical Centre Ljubljana, 1000 Ljubljana, Slovenia

**Keywords:** coronary sinus reducer, refractory angina, oxygen consumption

## Abstract

Coronary sinus reducer (CSR) implantation is a new treatment option for patients with refractory angina pectoris. However, there is no evidence from a randomized trial that would show an improvement in exercise capacity after this treatment. The aim of this study was to evaluate the influence of CSR treatment on maximal oxygen consumption and compare it to a sham procedure. Twenty-five patients with refractory angina pectoris (Canadian Cardiovascular Society (CCS) class II–IV) were randomized to a CSR implantation (*n* = 13) or a sham procedure (*n* = 12). At baseline and after 6 months of follow-up, the patients underwent symptom-limited cardiopulmonary exercise testing with an adjusted ramp protocol and assessment of angina pectoris using the CCS scale and Seattle angina pectoris questionnaire (SAQ). In the CSR group, maximal oxygen consumption increased from 15.56 ± 4.05 to 18.4 ± 5.2 mL/kg/min (*p* = 0.03) but did not change in the sham group (*p* = 0.53); *p* for intergroup comparison was 0.03. In contrast, there was no difference in the improvement of the CCS class or SAQ domains. To conclude, in patients with refractory angina and optimized medical therapy, CSR implantation may improve oxygen consumption beyond that of optimal medical therapy.

## 1. Introduction

A coronary sinus reducer (CSR) is a novel percutaneous treatment option for patients with refractory angina pectoris (RA) who are not amenable to further revascularization via percutaneous intervention or coronary artery bypass grafting (CABG) [1,2]. The hourglass-shaped design of the CSR device creates a focal narrowing in the distal coronary sinus, which presumably increases backward venous pressure and restores the perfusion ratio between the ischemic subendocardial and non-ischemic subepicardial myocardium [3].

While treatment with CSR is available in Europe, it has not yet been approved in the United States. To date, only one randomized trial evaluating the effectiveness of treatment with CSR was published (COSIRA study) [4]. While this study showed a significant improvement in subjective measures, such as the Canadian Cardiovascular Society (CCS) class and quality of life, it failed to confirm the effect of CSR implantation on the objective improvement of exercise capacity as assessed by symptom-limited exercise stress testing. Some non-randomized studies and registry reports have since reported increased walking distance for the 6 min walk test and increased exercise time following CSR implantation [5,6]. To date, only one study showed the improvement of oxygen kinetics using cardiopulmonary exercise testing (CPET) after CSR [7]. However, these patients were not randomized, and no sham procedure was performed, leading to potential bias due to the known placebo effect [8].

The aim of our study was therefore to evaluate the influence of CSR treatment on maximal oxygen consumption and compare it to a sham procedure.

## 2. Materials and Methods

This was an investigator-initiated, single-center, randomized, double-blind, sham-controlled study that included patients undergoing CSR implantation at University Medical Centre Ljubljana, Slovenia, between 1 January 2019 and 31 December 2021. Eligible patients had RA with Canadian Cardiovascular Society (CCS) class II–IV despite optimal medical therapy (OMT) for at least 30 days and reversible ischemia in the left anterior descending (LAD) and/or left circumflex (LCX) coronary artery confirmed via single photon emission tomography (SPECT), quantified as summed difference score (SDS) between stress and rest and the percentage (%) of left ventricle mass exhibiting reversible ischemia (performed and evaluated by L.L.). Exclusion criteria were non-stable angina pectoris within the last month, acute myocardial infarction within 3 months, successful revascularization by PCI or CABG within 6 months, decompensated heart failure, severe valvular heart disease, and co-morbidities known to preclude exercise stress testing.

Included patients were randomized to either CSR implantation (Neovasc Inc., Richmond, BC, Canada) or a sham procedure. Randomization was performed by M.M. and not disclosed to other investigators during the study. CSR implantation followed the standard technique that has been extensively described elsewhere [9]. A sham procedure included venous puncture, catheterization of the right internal jugular vein using the same 9 Fr introducer, and right atrium pressure measurement. All procedures were performed in the same catheterization laboratory and by the same experienced operator (M.B.). To ensure the best possible blinding, both CSR implantations and sham procedures were performed in auditory isolation provided by music played over headphones.

A symptom-limited cardiopulmonary exercise stress test (CPET) on a cycle ergometer (Cardiovit CS 200 Excellence ErgoSpiro, Schiller, Baar, Switzerland) using an adjusted ramp protocol was performed at baseline and after 6 months. The exercise protocol was individually adjusted to the estimated exercise capacity calculated by the Wasserman equation to ensure comparable exercise times and followed the warming-up period of 2 min. After 6 months, the CPET was repeated during the same time of day in the same environment and using the same exercise protocol. All the participants and the medical personnel performing CPET (S.C. and D.L., see Acknowledgments) remained blinded and unaware of patient allocation throughout the study.

Angina severity and quality of life (QoL) were assessed with CCS score and Seattle angina questionnaire (SAQ) at baseline and 6 months after the procedure. Clinical evaluation and CCS grading were performed during outpatient visit by physician blinded to the patient allocation (N.P.), while SAQ was completed by each patient alone. CCS class was graded on a scale of 1 to 4 depending on the clinical information provided by the patient, while the SAQ score was calculated for each of the 5 domains separately (physical limitation, angina stability, angina frequency, treatment satisfaction, QoL).

Categorical variables are represented as frequencies and percentages and were compared using chi-square and Fisher exact tests as appropriate. Continuous variables are presented as mean (standard deviation [SD]) or as median (interquartile range [IQR]). Normality of distribution was tested with the Kolmogorov–Smirnov test. Intra- and intergroup differences were compared with the use of independent or paired sample Student *t*-test, Wilcoxon rank-sum test, and Wilcoxon signed-rank test as appropriate. A two-sided *p*-value of 0.05 was considered statistically significant. Statistical analysis was performed in IBM SPSS Statistics for Windows, version 22.0 (IBM Corp., Armonk, NY, USA).

The CROSSROAD study was approved by the National Ethics Committee, and all the patients signed written consent. The study protocol and the letter of approval are available as supplements. The patients randomized to the sham procedure were offered CSR implantation after the completion of follow-up. (ClinicalTrials.gov: NCT04121845)

## 3. Results

**Figure 1:**
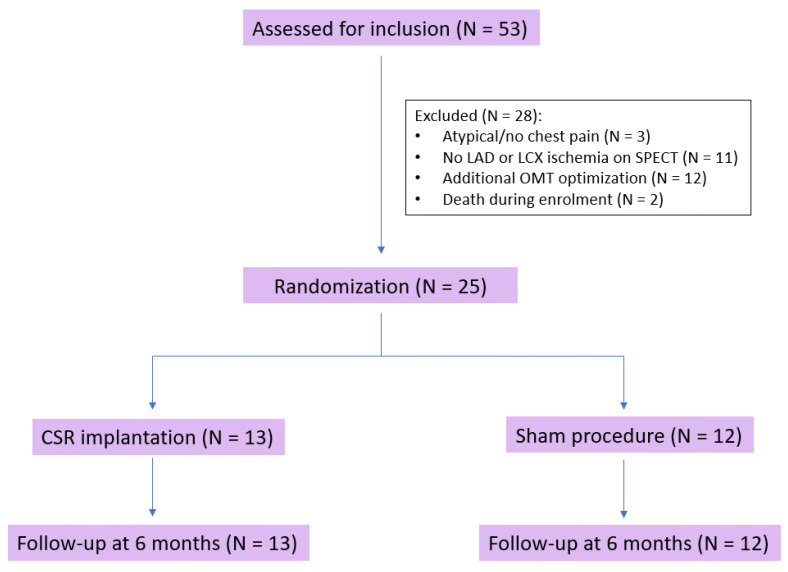
Flow diagram. Abbreviations: CSR—coronary sinus reducer, LAD—left anterior descending coronary artery, LCX—left circumflex coronary artery, OMT—optimal medical therapy.

Fifty-three patients were evaluated for study inclusion. Three patients (5.7%) were excluded due to atypical or no chest pain and eleven (20.8%) due to lack of LAD or LCX ischemia demonstrated by SPECT. Twelve patients (22.6%) were excluded after additional OMT optimization, and two (3.8%) died during enrollment. Therefore, twenty-five patients (84% male, aged 70.1 ± 10.8 years) underwent randomization and were included in the final analysis (Figure 1).

Thirteen patients were randomized to CSR implantation (treatment group) and twelve to the sham procedure (control group). All patients had advanced coronary artery disease with previous percutaneous (PCI) (64%) or surgical (84%) revascularization. The majority of patients had 3-vessel disease, and more than 75% had chronic total occlusion (CTO) of at least 1 coronary artery. Before inclusion, unsuccessful CTO revascularization was attempted in four patients randomized to the treatment group and six patients randomized to the sham procedure. In the remaining patients, CTO lesions were not considered suitable for PCI due to unfavorable anatomy (ostial and distal lesions, small vessel caliber, heavily calcified lesions) and limited area of ischemia on SPECT or diffuse coronary artery disease. Baseline characteristics, including CPET parameters, CCS class, and SAQ, did not differ between groups (Table 1). Optimal medical therapy was optimized in both groups and did not change during follow-up.

**Figure 2:**
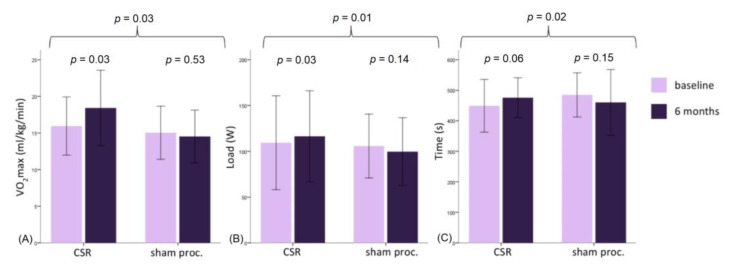
Maximal oxygen consumption (VO2 max) (**A**), maximal load (**B**), and exercise time (**C**) at baseline and 6-month follow-up. Abbreviations: CSR—coronary sinus reducer.

At the 6-month follow-up, maximal oxygen consumption increased in the treatment group (+2.46 ± 3.30 mL/kg/min, *p* = 0.03) but did not change in the control group (−0.52 ± 2.78 mL/kg/min, *p* = 0.53); *p*-value for intergroup comparison was 0.03. (Figure 2) This was consistent with the maximal load increase in the treatment but not in the control group (*p* = 0.01). The respiratory exchange ratio at both baseline and follow-up testing was high and constant, demonstrating maximal patient effort. Intra- and intergroup comparisons showed no difference in other CPET parameters—oxygen pulse, anaerobic threshold, dVO2/dWR, and VE/VCO2 (Table 2).

**Figure 3:**
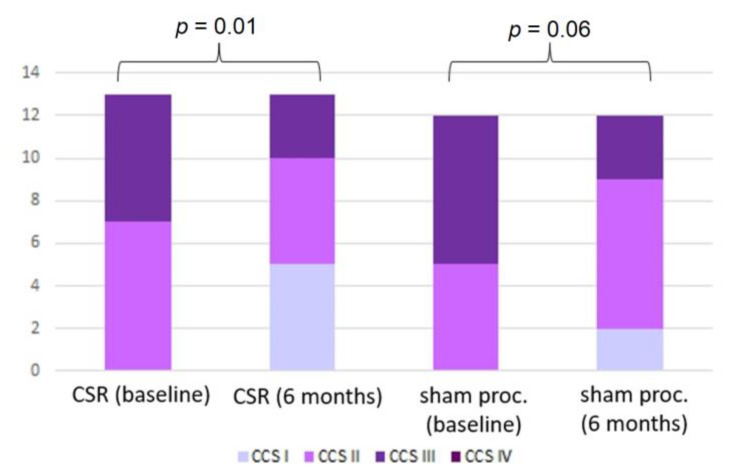
Distribution of Canadian Cardiovascular Society (CCS) angina class at baseline and 6-month follow-up. Abbreviations: CSR—coronary sinus reducer group, sham proc.—sham procedure group.

While the CCS class improved in the treatment group (*p* = 0.01), the improvement in the control group did not reach statistical significance (*p* = 0.06) (Figure 3). At follow-up, CSR patients reported improved physical limitations (*p* < 0.01), angina frequency (*p* < 0.01), and QoL (*p* = 0.01) domains of the SAQ. After the sham procedure, patients reported improved angina stability (*p* = 0.04), frequency (*p* = 0.02), QoL (*p* = 0.02), and treatment satisfaction (*p* = 0.02) (Table 3). However, in contrast to the difference in exercise parameters, there was no intergroup difference in the improvement of either the CCS class or any SAQ domain.

## 4. Discussion

To the best of our knowledge, this is the first randomized, sham-controlled study to show that CSR could improve exercise capacity beyond that of optimized anti-anginal medical therapy. In contrast to oxygen consumption, the improvement of subjectively assessed angina symptoms did not differ between both groups.

As all our patients were receiving optimized medical therapy before enrollment, no patients in CCS class IV were included. Despite this, the symptomatic status of the included patients is evident from the low baseline oxygen consumption. The observed values are lower than in other angina trials but comparable to the only previous CSR study exploring oxygen kinetics and to COSIRA patients if we compare the reported metabolic equivalents (METs) [4,7,10].

In the COSIRA trial, an asymptomatic cardiac ischemia pilot (ACIP) treadmill exercise protocol was used, which is a stage incremental protocol (1.5 METs/stage) [11]. Researchers reported no significant difference in exercise duration, achieved METs, or time to ST segment depression between the CSR and the control group. In contrast, our study used maximal, symptom-limited CPET for exercise capacity evaluation. Compared to standard ECG treadmill exercise testing, CPET provides additional information about the cardiopulmonary response to exercise and improves diagnostic accuracy for detection of myocardial ischemia [12,13]. The adjusted ramp protocol enables individual workload adaptation and is characterized by a linear and continuous increase of load, which avoids brisk step increases [14,15]. Furthermore, adaptation of the protocol to the individual exercise capacity resulted in a lesser degree of interindividual variation in exercise time. As in the COSIRA trial, double products at maximal exercise were low, which is the result of low exercise capacity and carefully optimized medical therapy. Thus, due to the low sensitivity, the time to ST depression may not be a relevant outcome in refractory angina patients.

Zivelonghi et al. reported improvements in maximal oxygen consumption and maximal exercise load following CSR implantation [7]. However, in contrast to our study, it was a multi-center registry study that lacked a control group. To minimize the pronounced placebo effect associated with invasive procedures, we compared the treatment group with a sham procedure. The subjective measures of angina pectoris, including quality of life, improved in both our groups and did not differ at follow-up. Baseline and follow-up SAQ scores of all angina pectoris domains were comparable to patients enrolled in the larger COSIRA trial. In line with our results, the investigators of the COSIRA trial did not find any intergroup difference in the improvement of SAQ with the exception of the quality of life domain, which showed a marginal statistical difference in favor of the treatment group (*p* = 0.048) [11]. This could be explained by the known placebo effect, which is even more pronounced after invasive procedures and interventions for pain-related conditions [8,16]. To the contrary, exercise parameters assessed by CPET improved only in patients treated with CSR but not in the control group. Our findings underline the importance of a sham control and evaluation of objective exercise capacity parameters, as they are less prone to subjective assessment than symptomatic, score-based measures that were used in most previous CSR studies.

Our study evaluated the efficacy of CSR in patients who were already treated with guideline-directed medical therapy. On average, patients were receiving at least three anti-ischemic agents which were continued throughout the study period and were not discontinued before the CPET. In contrast, larger trials exploring the efficacy of medical treatment either tested the medical agent as monotherapy or allowed the washout period before exercise testing [17,18,19]. Comparison of our results with these older trials is further restricted by the use of different exercise protocols, as these trials used mainly the modified Bruce or staged cycloergometric protocols, making direct comparisons of exercise times impossible.

The main limitation of our study is the relatively small number of included patients, which is the result of its single-center design and strict inclusion criteria, especially the need for fully optimized medical therapy and demonstrable ischemia on SPECT. With the increasing implantation rates of CSR and possible expanding indications, the results should be confirmed in a larger, multi-center clinical trial.

To address the ethical aspect of the study, the authors would like to disclose that after the completion of the follow-up, the CSR was implanted in seven patients randomized to the sham procedure. One patient did not decide on implantation due to a newly diagnosed non-cardiac disease, one patient underwent unsuccessful implantation due to the coronary sinus valve precluding the implantation procedure, and three patients decided to continue with medical therapy alone.

In conclusion, the randomized, sham-controlled CROSSROAD study demonstrated that in patients with RA and optimized medical therapy, CSR implantation may improve oxygen consumption beyond that of optimal medical therapy.

## Figures and Tables

**Table 1:** Baseline parameters. CSR—coronary sinus reducer group, Sham—control group which underwent a sham procedure. *p* ^†^ for intergroup comparison.

	CSR (*n* = 13)	Sham (*n* = 12)	*p* ^†^
Age—years ± SD	70.3 ± 10.3	69.8 ± 11.7	0.92
Male—*n* (%)	11 (84.6%)	10 (83.3%)	1
Hyperlipidemia—*n* (%)	13 (100%)	11 (91.7%)	0.48
Arterial hypertension—*n* (%)	13 (100%)	10 (83.3%)	0.22
Diabetes—*n* (%)	3 (23.1%)	4 (33.3%)	0.67
oGF—mL/kg/min ± SD	62.1 ± 16.1	66.6 ± 14.0	0.47
Previous PCI—*n* (%)	7 (53.8%)	9 (75%)	0.41
Previous CABG—*n* (%)	12 (92.3%)	9 (75%)	0.32
X-vessel disease—*n* (%)			
1	0	2 (16.7%)	0.22
2	3 (23.1%)	2 (16.7%)	1.0
3	10 (76.9%)	8 (66.7%)	0.67
Chronic total occlusion—*n* (%)	10 (76.9%)	9 (75%)	1.0
Ischemia distribution			
Anterior	6 (46.2%)	7 (58.3%)	0.54
Anterolateral	6 (46.2%)	5 (41.7%)	1.0
Inferolateral	7 (53.8%)	5 (41.7%)	0.54
Inferior	3 (23.1%)	2 (16.7%)	1.0
Septal	3 (23.1%)	1 (8.3%)	0.6
Reversible ischemia (%)	8.92 ± 7.1	8.82 ± 6.06	0.97
Summed difference score	6.92 ± 6.8	6.27 ± 5.75	0.81
Anti-ischemic therapy			
Beta blocker—*n* (%)	13 (100%)	12 (100%)	1
Ca antagonist—*n* (%)	2 (15.4%)	3 (25%)	0.65
Nitrate—*n* (%)	4 (33.3%)	7 (58.3%)	0.18
Trimetazidine—*n* (%)	13 (100%)	10 (83.3%)	0.22
Ranolazine—*n* (%)	10 (76.9%)	12 (100%)	0.22
Ivabradine—*n* (%)	2 (15.4%)	0	0.48
No. of drugs per patient—mean ± SD	3.31 ± 0.75	3.67 ± 0.78	0.32
CCS (baseline)			
II—*n* (%)	7 (53.8%)	5 (41.7%)	
III—*n* (%)	6 (46.2%)	7 (58.3%)	
IV—*n* (%)	0	0	0.54

Abbreviations: Ca—calcium, CABG—coronary artery bypass grafting, CCS—Canadian Cardiovascular Society score, CSR—coronary sinus reducer, PCI—percutaneous coronary intervention, SD—standard deviation.

**Table 2:** Baseline and follow-up CPET parameters. CSR—coronary sinus reducer group, Sham—control group which underwent a sham procedure. *p* ^†^ for intergroup comparison, *p* * for intragroup comparison of CPET parameters at baseline and 6 months after the procedure.

	CSR (*n* = 13)		Sham (*n* = 12)		*p* ^†^
CPET (baseline)					
Oxygen consumption -ml/kg/min ± SD	15.56 ± 4.05		15.04 ± 3.63		0.74
Workload—W ± SD	107 ± 50		106 ± 35		0.94
Time—s ± SD	445 ± 84		485 ± 72		0.23
Respiratory exchange ratio (±SD)	1.12 ± 0.15		1.13 ± 0.13		0.85
Anaerobic threshold (IQR)	11.3 (8.3–12.8)		9 (8.4–11.1)		0.46
Oxygen pulse—ml/beat ±SD	11.6 ± 5.1		13.2 ± 2.9		0.37
Double product (±SD)	19,292 ± 4539		16,710 ± 4390		0.16
VE/VCO2 (±SD)	28.6 ± 6.2		28.9 ± 3.5		0.88
dVO2/dWR (IQR)	8.2 (6.2–11.2)		7.9 (6.6–11.7)		1
CPET (6 months)		*p* *		*p* *	
Oxygen consumption—mL/kg/min ± SD	18.4 ± 5.2	0.03	14.52 ± 3.61	0.53	0.03
Workload—W ± SD	116 ± 50	0.03	100 ± 37	0.14	0.01
Time—s ±SD	467 ± 74	0.06	460 ± 107	0.15	0.02
Respiratory exchange ratio (±SD)	1.11 ± 0.12	0.64	1.09 ± 0.14	0.38	0.59
Anaerobic threshold (±SD)	12.4 ± 2.9	0.15	10.2 ± 1.7	0.96	0.36
Oxygen pulse—mL/beat ±SD	12.9 ± 4.8	0.31	13.5 ± 3.5	0.6	0.58
Double product (±SD)	18600 ± 4793	0.41	15968 ± 3693	0.48	0.89
VE/VCO2 (±SD)	28.0 ± 5.7	0.79	27.7 ± 7.6	0.39	0.76
dVO2/dWR (±SD)	9.3 ± 2	0.8	8.7 ± 2	0.84	0.82

Abbreviations: CPET—cardio-pulmonary oxygen testing, CSR—coronary sinus reducer, IQR—interquartile range, SD—standard deviation.

**Table 3:** Baseline and follow-up Seattle Angina Questionnaire (SAQ) score. CSR—coronary sinus reducer group, Sham—control group which underwent a sham procedure. *p* ^†^ for intergroup comparison, *p* * for intragroup comparison of SAQ score at baseline and 6 months after the procedure.

	CSR (*n* = 13)		Sham (*n* = 12)		*p* ^†^
SAQ (baseline)					
Physical limitation (±SD)	34.6 ± 12		29.6 ± 15.3		0.38
Angina frequency (±SD)	37.8 ± 21.1		40.9 ± 23.9		0.73
Angina stability (IQR)	40 (40–40)		40 (5–40)		0.54
Quality of life (IQR)	33.3 (23.4–36.7)		33.4 (8.4–45)		0.98
Treatment satisfaction (IQR)	57.1 (47.6–71.4)		57.1 (50.0–61.9)		0.69
SAQ (6 months)		*p* *	*p* *		
Physical limitation (±SD)	46.9 ± 10.6	<0.01	37.5 ± 17.6	0.07	0.59
Angina frequency (±SD)	59.0 ± 15.7	<0.01	59.8 ± 21.3	0.02	0.84
Angina stability (±SD)	50.0 ± 20.0	0.11	47.3 ± 16.2	0.04	0.84
Quality of life (±SD)	44.4 ± 10.4	0.01	47.9 ± 24.9	0.02	0.54
Treatment satisfaction (±SD)	64.7 ± 9.4	0.19	63.2 ± 9.3	0.02	0.43

## Data Availability

The data presented in this study are available on request from the corresponding author.
